# Perspective: Present and Future of Virtual Reality for Neurological Disorders

**DOI:** 10.3390/brainsci12121692

**Published:** 2022-12-09

**Authors:** Hyuk-June Moon, Sungmin Han

**Affiliations:** 1 Bionics Research Center, Biomedical Research Division, Korea Institute of Science and Technology (KIST), Seoul 02792, Republic of Korea; 2 Division of Bio-Medical Science & Technology, Korea Institute of Science and Technology School, Korea University of Science and Technology (UST), Seoul 02792, Republic of Korea

**Keywords:** virtual reality, neurological, cognitive, rehabilitation, diagnosis, metaverse

## Abstract

Since the emergence of Virtual Reality technology, it has been adopted in the field of neurology. While Virtual Reality has contributed to various rehabilitation approaches, its potential advantages, especially in diagnosis, have not yet been fully utilized. Moreover, new tides of the Metaverse are approaching rapidly, which will again boost public and research interest and the importance of immersive Virtual Reality technology. Nevertheless, accessibility to such technology for people with neurological disorders has been critically underexplored. Through this perspective paper, we will briefly look over the current state of the technology in neurological studies and then propose future research directions, which hopefully facilitate beneficial Virtual Reality studies on a wider range of topics in neurology.

## 1. Introduction: Present of Virtual Reality

**Figure 1:**
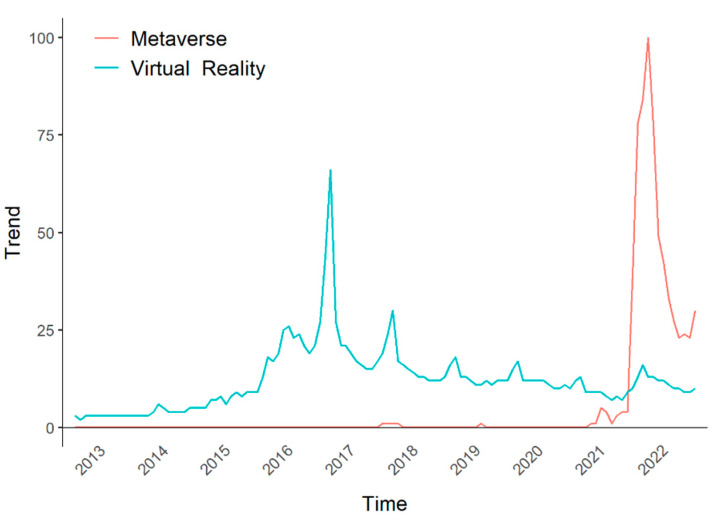
The results of Google Trends for ‘Metaverse’ and ‘Virtual Reality’ over time (2013 to 2022). The trends show the Virtual Reality boom in the mid-2010s and the recent emergence of the Metaverse.

In the mid-2010s, with the launches of several commercial products, Virtual Reality (VR) technology attracted significant public and research attention (although, surprisingly, the origin of the technology dates back to the 1980s) [1,2]. Thanks to its immersive system, including head-mounted displays (HMDs), it has an outstanding capability to simulate lifelike experiences from the first-person perspective (1 PP). Thus, VR has been introduced to the field of psychiatric disorders, for instance, as therapy for phobias, anxiety disorders, and post-traumatic stress [3,4,5]. Likewise, VR has been utilized in the rehabilitation of patients with neurological disorders, motivating them to be more actively engaged and immersed via fun and game-like methods (for a review [6,7]). While VR technology has been shown to facilitate recovery and enhance motor or cognitive functions in patients with Alzheimer’s disease (AD), Parkinson’s disease (PD), or stroke, it has rarely been used for the diagnosis of such diseases or the evaluation of the related cognitive impairments, where it has many advantages over conventional methods. In contrast to the exaggerated expectations of the time when it first appeared, the era of VR (replacing other traditional displays) has not immediately arrived due to many obstacles: lack of affordable consumer products and enjoyable content, difficulties in dedicated software development, limited hardware performance (e.g., insufficient computing power and display resolutions, usage with bulky cables, or limited battery time), limited sensory modalities, and cybersickness (nausea, vomiting, dizziness, or fatigue) [8,9]. Along with public trends, it has also become less actively adopted in research than before, including the field of neurology. However, those obstacles are decreasing thanks to the continuous development of better and more affordable VR systems, and, at last, the recent rise of the Metaverse is about to bring the second golden age of VR technology (Figure 1).

At this very critical moment, through this short communication, we would like to propose ways to better utilize advanced VR technology for patients with neurological disorders (focusing on diagnosis and evaluation) and would also like to suggest future research avenues necessary for such patients to fully enjoy the era of the Metaverse together. Hopefully, this can facilitate various VR studies to benefit neurological patients. Of note, in this manuscript, VR refers to immersive VR with a stereo HMD [2], rather than the too broad definition of ‘a computer-generated world’.

## 2. Possibility for Diagnosis or Assessment of Cognitive Impairments

Many neurological diseases accompany a wide range of cognitive impairments, which can lead to mild cognitive impairment (MCI) and even to dementia at later stages [10,11]. AD is the most common cause of dementia, and there are also other neurological diseases that can significantly impair cognitive functions, such as stroke, PD, and dementia with Lewy bodies. Various kinds of tests (for instance, the Mini-Mental State Examination (MMSE), the Montreal Cognitive Assessment (MoCA), and Mini-Cog) are currently used to assess cognitive impairments and screen such diseases [12,13]. However, those tests are often not straightforward to interpret: their results are affected by education level, language, and race/ethnicity [12,14]. Moreover, there can be ceiling or floor effects for scoring an individual category of cognitive functions, and the summed binary or ordinal score data are often analyzed as metric data, which might lead to statistically incorrect results [15]. Due to such issues, it is difficult for such tests to be used generally to diagnose various diseases and to distinguish different stages of their progression (e.g., MCI from dementia).

**Figure 2:**
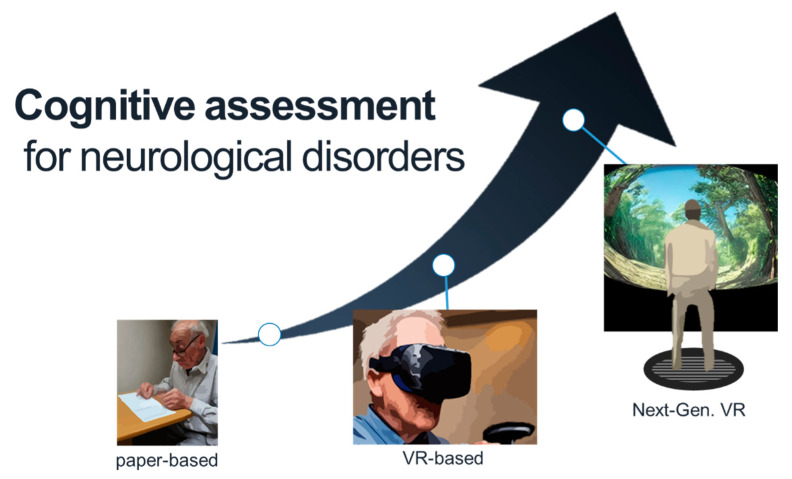
Cognitive assessment tools for neurological disorders will hopefully evolve from paper-based tests into next-generation VR-based tests with advances in VR technology.

Hopefully, VR-based tests can serve as better assessment tools for cognitive impairments (Figure 2). First, VR can simulate situations that may occur in our daily lives (e.g., buying a list of products at the market or navigating to specific places in town) and assess the relevant cognitive abilities (e.g., working memory, executive function, spatial navigation, spatial memory, and cognitive planning) as a VR-based cognitive test. The results of such tasks can directly indicate whether subjects can adequately perform the specific tasks in their lives (so that they can request support if needed). Notably, the likely situations are shown in the 1PP through VR systems (including HMDs and sound systems), which dramatically boosts immersion in the virtual scenario (i.e., the cognitive test) and, arguably, makes the test results even more reliable.

In addition, not only the mere binary results of the task (i.e., success or failure) but also many detailed parameters, which might contain much richer information regarding one’s cognitive functions, can be acquired during VR-based tasks. For instance, Kunz and colleagues [16] reported navigation pattern changes (i.e., a tendency to navigate closer to arena boundaries) during a spatial navigation memory task in a group with a genetic risk of AD, while their spatial memory precision did not differ from the healthy control group (arguably due to the compensatory mechanisms of intact brain regions). Likewise, many other parameters of VR tasks (e.g., navigation distance or trial time [17]) may convey information about disease-related cognitive impairments that could be missing from conventional tests.

Importantly, VR-based tests can provide unbiased and more objective test results than conventional methods (especially those using traditional displays). In VR paradigms, a virtual experimenter instead of a human instructor can give instructions, reducing any influence or bias possibly introduced by different experimenters and improving double-blind or multi-center experimental designs [18]. In addition, VR can fully control visual inputs, on which human beings largely depend [19]. HMDs can mask other visual inputs to minimize possible distractions during tests. Moreover, the size of any text or object shown to participants can be precisely controlled by stereoscopic HMDs (whereas for traditional displays, diverse screen sizes and the distance from the screen can critically affect those parameters). The control of visual input will be even more critical if cognitive tests are combined with neuroimaging since differences in visual information can significantly affect one’s brain activity [20]. This can help standardize multi-center research or even allow home-based tracking of patients’ cognitive abilities, which will likely promote the neurorehabilitation of life-relevant functions through repetitions of such VR tasks [6,7]. Additionally, VR with HMDs can be used to detect decreased stereopsis in PD patients [21], providing more standardized and controlled assessment methods.

## 3. Next-Generation VR Systems

More advanced VR systems combined with technologies from other fields (for instance, robotics, neuroscience, or materials) are promising candidates for next-generation VR paradigms that can benefit patients with neurological disorders. A recently published study by Alberts and colleagues integrated a VR system with an omnidirectional treadmill and implemented a VR grocery shopping task [22], which allowed them to assess patients’ cognitive-motor functions (beyond just cognitive ones) with various detailed behavioral parameters during the task (e.g., the occurrence of freezing of gait). This study showed significant progress toward ideal VR-based testing. Such a paradigm can further assess the dual-task (or multiple-task) abilities of patients [23]. It has been shown that performing physical and cognitive tasks simultaneously (as is usually required in our daily lives) can significantly worsen individual task performance, especially when each demands a high level of attention. As expected, decreased postural control or gait performance has been reported in the elderly when presented with dual-task situations (who need to pay more attention than the young population when performing such physical tasks, as do patients with neurological disorders) [24]. Hence, such VR-motor paradigms can more accurately evaluate a patient’s real-life abilities. However, despite the advantages of the VR-motor platform, the cost of the omnidirectional treadmill used in the study (Infinadeck, starting at USD 40 K according to the manufacturer’s website) is not easily affordable for many clinics or research institutes (and even by individuals who can benefit from home-based VR approaches). There are also alternative omnidirectional VR treadmills with more affordable prices (USD 1~2 K), such as KAT VR or Omni One. Thus, more studies should follow to investigate whether those devices (which seem to target young people playing VR games) can also be applied to elderly individuals, especially those with various neurological conditions, in terms of usability and safety.

To develop and establish a next-generation VR-based cognitive assessment, it is first necessary to design an appropriate VR-based task based on the real-life necessary abilities or cognitive (or cognitive-motor) functions to be tested. Importantly, as mentioned above, the task parameters should be standardized as much as possible through VR systems so that the data from various studies can be integrated without problems. Then, using a VR-based task, enough data should be collected from diverse populations with different ethnicities, races, sexes/genders, and diseases (necessarily through international collaborations). The collected data should be analyzed to establish appropriate methods to quantify each category of cognitive functions. Along the way, the quantified VR task results should also be compared with other tests currently used in clinics (e.g., the MMSE and the MoCA) to validate their utility and whether they can replace conventional tests at some point (after a broad consensus is formed).

Notably, there are novel VR-based neuroscience approaches that experimentally induce hallucinations similar to psychiatric symptoms, such as out-of-body experiences [25,26,27]. Importantly, a recent study by Bernasconi and colleagues demonstrated that a robot-based experimental induction of a psychiatric symptom could tell whether a PD patient was experiencing such a symptom in one’s daily life; presence hallucinations (from which up to 60% of PD patients suffer [28]) were more easily induced in the lab for patients with the same symptoms [29]. Such approaches combining VR and cognitive neuroscience can be further applied to diagnose neurological diseases with psychiatric symptoms (e.g., hallucinations and delusions) and to predict related prognoses.

## 4. The Metaverse for Patients with Neurological Disorders

We are now seeing the rise of the Metaverse (Figure 1). The term ‘Metaverse’ was first introduced in the science fiction novel *Snow Crash* (1992) as an immersive virtual world that users can access via a VR system [30]. Importantly, in the Metaverse, people can live as an avatar (computer-generated body, which does not necessarily resemble one’s physical body) and interact with others (i.e., avatars of other people) [31]. Thanks to the advent of the internet and advanced VR systems, the Metaverse, which was only imagined three decades ago, is now about to be realized. Public and research interest in it is rapidly increasing. The Metaverse is often linked to immersive experience in virtual worlds. Accordingly, interest in VR systems that could provide a more realistic and diverse sensory experience than conventional devices is also growing again. Of note, the Metaverse can be especially beneficial for patients with neurological disorders, allowing them to freely move around and communicate with others in the virtual world, unlike in the real world. This can benefit not only patients’ social/mental health (as most of us experienced during the COVID-19 pandemic [32]) but also their cognitive functions [33]. However, the digital divide, which exists now for the use of digital devices or online/internet services [34,35] and will probably become more critical for VR devices and the Metaverse, needs to be addressed so that the elderly and especially patients with neurological diseases do not fall behind. Such patients likely have brain mechanisms or functions that differ from those of the healthy population. Hence, it should be investigated whether they can access immersive VR and the Metaverse without suffering from severe cybersickness or fatigue. Furthermore, studies on how to prevent or ameliorate such VR-induced side effects, such as whether continued exposure to an immersive VR experience may alleviate or exacerbate them or whether physiological stimulation (e.g., on the vestibular system or brain) can help, should also be followed.

## 5. Conclusions

When VR was first introduced in the field of neurology, it was expected to open a new stage of clinical research. However, the advantages of VR have yet to be fully utilized. While the rapidly approaching tides of the Metaverse can benefit patients with neurological disorders, they will, at the same time, require them to be prepared and adapted to life in the new era, which can be even more challenging for these patients than for a healthy population. Hopefully, this paper will facilitate many future VR studies that benefit patients, providing better tools for assessing their cognitive impairments and enabling them to enjoy freer and happier lives in the Metaverse.

## Data Availability

Not applicable.
